# Mitogenomes Provide Insights into the Species Boundaries and Phylogenetic Relationships among Three *Dolycoris* Sloe Bugs (Hemiptera: Pentatomidae) from China

**DOI:** 10.3390/insects15020134

**Published:** 2024-02-17

**Authors:** Chenguang Zheng, Xiuxiu Zhu, Ying Wang, Xue Dong, Ruijuan Yang, Zechen Tang, Wenjun Bu

**Affiliations:** Institute of Entomology, College of Life Sciences, Nankai University, 94 Weijin Road, Tianjin 300071, China; chenguangzheng@nankai.edu.cn (C.Z.); 15376155087@163.com (Y.W.); dongxuer0123@163.com (X.D.); yrj1523@163.com (R.Y.); 2120201121@mail.nankai.edu.cn (Z.T.)

**Keywords:** *Dolycoris*, mitogenome, species delimitation, phylogeny

## Abstract

**Simple Summary:**

The three sloe bugs, *Dolycoris baccarum*, *D. indicus* and *D. penicillatus,* are distributed across the Chinese mainland and have similar morphology. In this study, the mitochondrial genomes of these three species were generated and compared. Species boundaries and interspecific relationships among the three species were explored for the first time. The mitochondrial genomes of the three *Dolycoris* species were conservative in terms of nucleotide composition, gene order, and codon usage. The species boundaries of the three species were clarified based on the mitochondrial data, and a clear barcode gap was found between the interspecific and intraspecific genetic distances. The interspecific relationships of the three species were inferred as (*D. indicus* + (*D. baccarum* + *D. penicillatus*)). Our study has enriched the mitogenomic library of *Dolycoris*, providing insights into the evolution of *Dolycoris* species.

**Abstract:**

(1) Background: The three sloe bugs, *Dolycoris baccarum*, *Dolycoris indicus,* and *Dolycoris penicillatus*, are found in the Chinese mainland and are morphologically similar. The species boundaries and phylogenetic relationships of the three species remain uncertain; (2) Methods: In this study, we generated multiple mitochondrial genomes (mitogenomes) for each of the three species and conducted comparative mitogenomic analysis, species delimitation, and phylogenetic analysis based on these data; (3) Results: Mitogenomes of the three *Dolycoris* species are conserved in nucleotide composition, gene arrangement, and codon usage. All protein-coding genes (PCGs) were found to be under purifying selection, and the ND4 evolved at the fastest rate. Most species delimitation analyses based on the COI gene and the concatenated 13 PCGs retrieved three operational taxonomic units (OTUs), which corresponded well with the three *Dolycoris* species identified based on morphological characters. A clear-cut barcode gap was discovered between the interspecific and intraspecific genetic distances of the three *Dolycoris* species. Phylogenetic analyses strongly supported the monophyly of *Dolycoris*, with interspecific relationship inferred as (*D. indicus* + (*D. baccarum* + *D. penicillatus*)); (4) Conclusions: Our study provides the first insight into the species boundaries and phylogenetic relationships of the three *Dolycoris* species distributed across the Chinese mainland.

## 1. Introduction

Mitochondrial genomes (mitogenomes) have been extensively used in the studies of species delimitation and phylogenetics [1,2,3,4,5,6]. The typical insect mitogenome is a double-strand circular molecule, ranging in size from 14 to 20 kb, with conserved gene arrangement and genetic composition [7,8]. Comparing the nucleotide composition, gene structure, and evolutionary rate of mitogenomes among species can provide important information related to their biological evolution [9,10,11]. In addition, mitochondrial gene sequences, specifically the animal DNA barcoding gene COI, are considered powerful molecular markers for delimiting species boundaries [12,13]. The single-locus barcoding approach can be of great help as an initial screening tool for species boundaries in poorly known taxa, or as a valuable complement to taxonomy in morphologically conserved taxa [14]. Furthermore, due to rare recombination, maternal inheritance, and a relatively high evolutionary rate, mitochondrial genomes are perceived as effective markers for resolving phylogenetic relationships in insects. The genome of this organelle has been widely used for both deeply divergent lineages and recently radiated groups [15,16].

**Figure 1:**
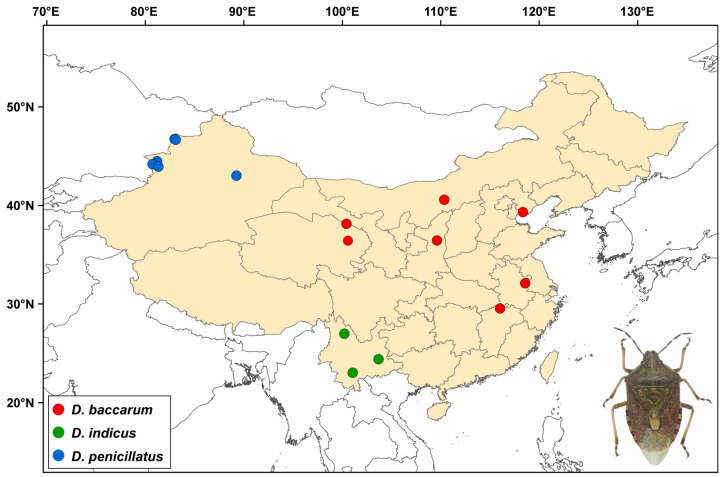
Geographical map of the *Dolycoris* sampling sites where material was collected in this study.

The genus *Dolycoris* Mulsant & Rey is mainly distributed in Eurasia, with 10 described species [17]. Among them, *Dolycoris baccarum* (Linnaeus), *Dolycoris indicus* Stål, and *Dolycoris penicillatus* Horváth are distributed across the Chinese mainland (Figure 1). These three species are similar morphologically but can be distinguished by the pygophore in male adults and the distribution regions [17]. Previous studies on these three species have mainly focused on life history, hazards posed, and prevention, especially for *D. baccarum*, a polyphagous pest of various crops, and one of the chief pests of alfalfa in China [18,19,20]. The genomics and evolution of these species have been poorly studied, with the exception of the mitochondrial genome (mitogenome) of *D. baccarum* that has been reported, and two mitogenomes of *D. baccarum* from different altitudes have been compared in detail [20]. However, the mitogenomes of *D. indicus* and *D. penicillatus* have never been studied, and the species boundaries and interspecific relationships among these three *Dolycoris* species are still unclear.

In this study, we generated multiple mitochondrial genomes for each of these three *Dolycoris* species. Based on these data, mitogenome comparison, species delimitation, and phylogenetic analysis were performed for the three species. Our aims are (1) to compare the feature of mitochondrial genome of the three species; (2) to preliminarily delimit the species boundaries of these species; and (3) to explore the phylogenetic relationships of these three *Dolycoris* species and the phylogenetic position of the genus *Dolycoris* within Pentatomidae.

## 2. Materials and Methods

### 2.1. Sampling, DNA Extraction, and Sequencing

A total of 15 *Dolycoris* specimens were sampled for mitogenome sequencing, representing three species from 15 locations in China (Table S1). Samples were collected from 2016 to 2022, preserved in 100% ethanol, and stored at −20 °C at the Institute of Entomology at Nankai University (Tianjin, China). We extracted the genomic DNA from the thoracic muscle using a Universal Genomic DNA Kit (CWBIO, Beijing, China). Whole mitochondrial genomes were sequenced individually using the 150 bp paired-end reads strategy via the Illumina NovaSeq 6000 platform. We used fastp [21] to remove low-quality reads. Finally, approximately two Gb of raw data was obtained for each individual.

### 2.2. Mitogenome Assembly and Annotation

The sequences of mitogenome were assembled using mitoZ 2.4 [22] with default settings and IDBA-UD [23], with k values ranging from 40 to 120 bp. Transfer RNA (tRNA) genes were annotated using the MITOS2 webserver (available at http://mitos2.bioinf.uni-leipzig.de/index.py, accessed on 2 April 2023) with invertebrate mitochondrial genetic code. Protein-coding genes (PCGs) and ribosomal RNA (rRNA) were annotated by alignment with the homologous genes of previously published mitochondrial genomes of *Dolycoris* baccarum in GenBank (accession numbers: NC_020373 and KJ507135). The annotated mitogenome sequences of 15 *Dolycoris* individuals were deposited in GenBank (Table S1).

### 2.3. Sequence Analyses

We used MEGA X [24] to calculate the nucleotide compositions of the whole mitogenome. The bias of the nucleotide composition for each mitogenome was measured by AT-skew [(A − T)/(A + T)] and GC-skew [(G − C)/(G + C)]. We also used MEGA X [24] to assess the codon usage of 13 PCGs. The non-synonymous substitution rate (Ka) and synonymous substitution rate (Ks) of each PCG was calculated using DnaSP 6.12.03 [25], and the ratio of Ka/Ks was used to represent the evolution rate of each PCG.

### 2.4. Species Delimitation

To conduct the preliminary exploration of the species boundaries of these three *Dolycoris* species, we performed four molecular methods of species delimitation based on the animal DNA barcoding gene COI and the concatenated 13 PCGs: Automatic Barcode Gap Discovery (ABGD) [26], Assemble Species by Automatic Partitioning (ASAP) [27], Bayesian Poisson Tree Processes (bPTP) [28], and Generalized Mixed Yule Coalescent model (GMYC) [29,30]. The concept of operational taxonomic units (OTUs) [31], which refers to the basic unit of classification in biodiversity science, was used to represent the species generated based on molecular data. ABGD analyses were conducted using the web server (https://bioinfo.mnhn.fr/abi/public/abgd/abgdweb.html, accessed on 14 April 2023) with these default settings (Steps = 10, X = 1.5, Pmin = 0.001, Pmax = 0.1, Nb bins = 20). The nucleotide substitution model of Kimura 2-P (K2P) was selected. ASAP analyses were conducted using the web server (https://bioinfo.mnhn.fr/abi/public/asap, accessed on 21 April 2023) with K2P nucleotide substitution model. The non-ultrametric trees as the input file for the bPTP analyses were reconstructed by IQ-TREE 2.2.0 [32] without outgroups. Then, bPTP analyses were performed on the web server (https://species.h-its.org/ptp/, accessed on 19 April 2023) with a Bayesian topology. The parameters were chosen to be 200,000 generations, a thinning of 100, and a burn-in of 10%. The ultrametric trees as the input file for GMYC analyses were reconstructed by BEAST 2.6.6 [33] without outgroups under a Yule model prior and a strict clock model. The Markov chain Monte Carlo (MCMC) chains were run for 1 million generations and sampled every 10,000 generations. We used Tracer 1.7 [34] to assess the convergence of runs. TreeAnnotator 2.6.6 [33] was used to generate consensus trees, and the first 10% of trees were discarded as burn-in. The GMYC analyses were conducted based on the ultrametric trees on the web server (http://species.h-its.org/gmyc/, accessed on 20 April 2023) under a single-threshold method. In addition, the intraspecific and interspecific genetic distances for each PCG were calculated using MEGA X [24] under the K2P nucleotide substitution model.

### 2.5. Phylogenetic Analyses

Phylogenetic analysis was performed using the 15 newly sequenced mitogenomes of the three *Dolycoris* species and 31 mitogenomes of Pentatomidae downloaded from GenBank (Table S2). Two species from the family Plataspidae, the sister family of Pentatomidae, were used as outgroups [35]. The 13 PCGs and two rRNA genes were aligned using the MAFFT algorithm at the EMBL-EBI [36] website (https://www.ebi.ac.uk/Tools/msa/mafft/, accessed on 25 April 2023). PhyloSuite 1.2.2 [37] was used to concatenate individual genes. Finally, we generated two datasets to reconstruct phylogenetic trees: PCG123R, a dataset containing three codon positions of the 13 PCGs and two rRNAs; and PCG12R, a dataset containing the first and second codon positions of the 13 PCGs and two rRNAs. The heterogeneity of sequence divergence within each dataset was analyzed using AliGROOVE 1.07 [38]. Phylogenetic analyses were carried out based on the two datasets using Bayesian inference (BI) and maximum likelihood (ML) methods. The best-fit substitution models and partitioning scheme were calculated using PartitionFinder 2.0 [39]. BI analysis was conducted by MrBayes 3.2.7a [40] with the most appropriate substitution model (Table S3). The Markov chain Monte Carlo (MCMC) method was used to calculate 10,000,000 generations, sampling every 1000 generations. The original 25% of trees were discarded as burn-in. The convergence of runs was checked by the deviation of split frequencies. ML analysis was conducted by IQ-TREE 2.2.0 [32], with 1000 ultrafast bootstraps under the best-fit substitution model.

## 3. Results

### 3.1. Mitogenome Features of the Three Dolycoris Species

**Figure 2:**
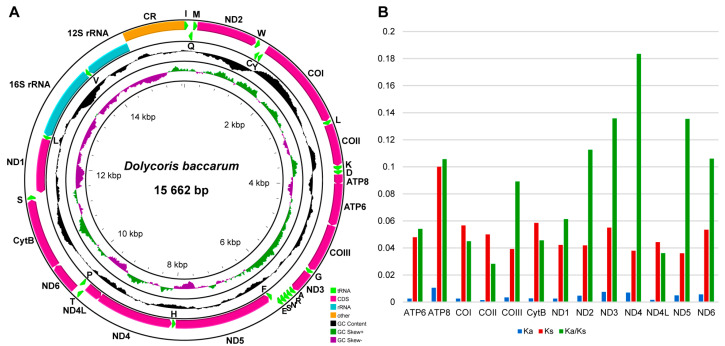
(**A**) Mitogenome map of the representative species of *Dolycoris* (*Dolycoris baccarum*). The direction of gene transcription is indicated by the arrows on the strands. PCGs and rRNAs are represented by normative abbreviations, while tRNAs are indicated by single−letter abbreviations. (**B**) Evolution rate of each PCG of the *Dolycoris* species.

The entire length of the 15 mitogenomes representing the three *Dolycoris* species ranged from 15,255 bp to 15,785 bp (Table 1). Each mitogenome contained 37 typical insect mitochondrial genes [7,8,20] (22 tRNAs, 13 PCGs, and 2 rRNAs,) and an A + T-rich region (control region) (Figure 2). Among the 37 genes, 14 tRNAs, 9 PCGs, and 2 rRNAs were located in the majority strand (J-strand), and the other 12 genes were located in the minority strand (N-strand). The entire sequences of these mitogenomes were A + T biased, and the A + T content of *D. baccarum*, *D. indicus,* and *D. penicillatus* was 73.2%, 73.0%, and 73.1%, respectively (Table 1). The values of AT-skew were positive (0.14–0.15), while the values of GC-skew were negative (−0.16–−0.15) in the 15 mitogenomes (Table 1). PCGs mainly started with ATN (ATA, ATT, ATG, and ATG), except for COI (TTG). The termination codons were TAA or truncated termination codon T (COI and COII) (Table S4). Each mitogenome contained 3675 codons, in addition to the termination codons. The most frequently used codon families were Ile, Leu2, and Met, while the least frequently used were Gln, Cys, and Arg (Figure S1). The value of Ka/Ks for each PCG was less than 0.2, ND4 exhibited the largest value of Ka/Ks, and COII had the lowest value of Ka/Ks (Figure 2).

### 3.2. Species Delimitation

**Figure 3:**
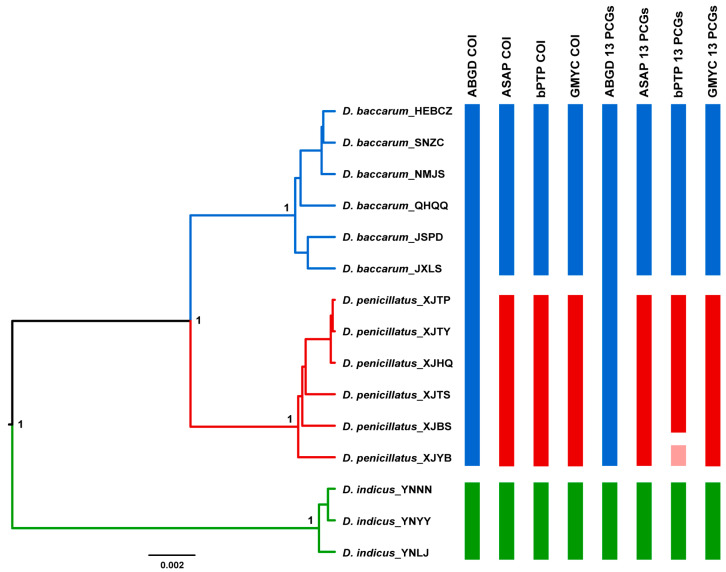
Summary of species delimitation results for the three *Dolycoris* species. The ultrametric tree on the left was reconstructed using BEAST based on the concatenated 13 PCGs. The columns on the right are the results of four delimitation methods based on molecular data (ABGD, ASAP, bPTP, and GMYC) based on the COI gene and the concatenated 13 PCGs. The putative species (OTUs) inferred by the molecular data are shown in different colors.

The ABGD method based on COI retrieved two OTUs when the prior intraspecific divergences (*p* values) varied from 0.0046 to 0.0129 (Figure 3). The individuals of *D. baccarum* and *D. penicillatus* were assigned to a single OTU, and the other OTU included all the *D. indicus* individuals (Figure 3). The ASAP analysis based on the COI gene produced three OTUs when the threshold distance was 0.007883, which is the best partition supported by asap-score and *p* value (Figure 3). The samples of each OTU were in accordance with the species identified according to their morphological characteristics. The bPTP and GMYC analyses based on COI supported the delimitation scenarios generated by ASAP (Figure 3). The delimitation results of ABGD, ASAP, and GMYC based on the concatenated 13 PCGs were consistent with those based on COI. The bPTP analysis based on the concatenated 13 PCGs yielded four OTUs, splitting *D. penicillatus* into two OTUs (Figure 3).

The intraspecific genetic distances of the three *Dolycoris* species were below 1% and differed among the 13 PCGs (Table 2). The intraspecific genetic distances based on the animal DNA barcoding gene COI were largest in *D. penicillatus* (0.28%), followed by *D. baccarum* (0.18%) and *D. indicus* (0.13%), while values based on the concatenated 13 PCGs were highest in *D. baccarum* (0.27%), followed by *D. penicillatus* (0.22%) and *D. indicus* (0.10%). The individual genes with the largest and smallest intraspecific genetic distances were different in the three species. The interspecific genetic distances between *D. baccarum* and *D. indicus*, between *D. baccarum* and *D. penicillatus*, and between *D. indicus* and *D. penicillatus* based on the COI gene were 2.91%, 1.55%, and 3.12%, respectively, indicating the relatively distant position of *D. indicus* and the close relationship between *D. baccarum* and *D. penicillatus*. Similar scenarios were generated based on the concatenated 13 PCGs and other individual genes, but the values varied among them.

### 3.3. Phylogenetic Analyses

**Figure 4:**
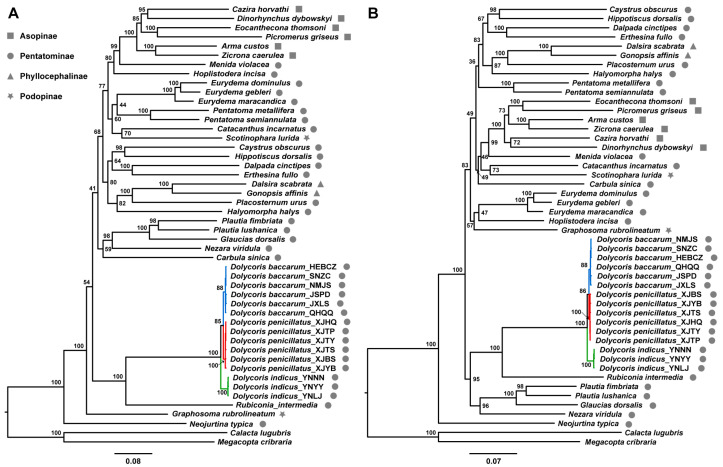
Phylogenetic trees of Pentatomidae inferred by ML analysis based on datasets PCG123R (**A**) and PCG12R (**B**). Numbers at the nodes are bootstrap values.

The heterogeneity of sequence divergence in the PCG123R dataset was close to that in the PCG12R dataset. For the datasets of PCG123R and PCG12R, the heterogeneity among the three *Dolycoris* species was lower than that between them and other species of Pentatomidae (Figure S2). The phylogenetic trees constructed by ML and BI analyses showed that Asopinae and Phyllocephalinae were highly supported as monophyletic, but the monophyly of Podopinae and Pentatominae was rejected. *Neojurtina typica* Distant was placed at the most basal position within Pentatomidae, and *Rubiconia intermedia* (Wolff) was recovered as a sister taxon of the three *Dolycoris* species in all trees (Figure 4 and Figure S3). The monophyly of *Dolycoris* was highly supported by all phylogenetic trees. Within *Dolycoris*, the ML trees reconstructed by the PCG123R and PCG12R datasets, and the BI tree based on the PCG12R dataset consistently supported the monophyly of each *Dolycoris* species and showed the same tree topology: (*D. indicus* + (*D. baccarum* + *D. penicillatus*)) (Figure 4 and Figure S3). However, the relationship of *D. indicus* as a sister of *D. baccarum* + *D. penicillatus* was not supported by the BI tree reconstructed by the PCG123R dataset (Figure S3).

## 4. Discussion

### 4.1. Mitogenome Features

The mitogenomes of *D. baccarum* from two different altitudes have been compared in detail [20], but the mitogenomes of its closely related species, *D. indicus* and *D. penicillatus*, have never been studied. Here, we sequenced 15 mitogenomes of three species of *Dolycoris* from 15 locations in China and performed a detailed comparative analysis. The entire length of the 15 mitogenomes varied slightly (15,255–15,785 bp), and the length difference is mostly due to the size of the control region, which is consistent with other Pentatomidae species [15]. The features of mitogenome of different individuals within the same species were basically consistent, including the nucleotide composition, gene composition, and codon usage of PCGs. The gene arrangements in the mitogenomes of the three *Dolycoris* species are conserved, consistent with the gene arrangement of the ancestral insect mitogenome [41]. The nucleotide composition of these species was biased toward A + T, which is in accordance with other mitogenomes of Pentatomidae species [42,43,44]. The three species of *Dolycoris* exhibit negative GC-skew and positive AT-skew in their mitogenomes, and the nucleotide bias in mitogenome may be associated with the asymmetric mutation processes during replication [45]. PCGs terminate primarily with TAA, with the exception of COI and COII, which terminate with a truncated termination codon T, possibly completed by post-transcriptional polyadenylation [46]. The codon usage is consistent among the three species of *Dolycoris*, with Ile being the most frequent codon family. The ratio of Ka/Ks is widely used to represent the evolutionary rate of PCGs in the mitogenome [11,47], and the low values of Ka/Ks for the 13 PCGs revealed in this study suggest that they are under purifying selection. Of the 13 PCGs, ND4 exhibits the largest value of Ka/Ks and COII exhibits the lowest, which is inconsistent with the other mitogenomes of Pentatomidae species, where COI and ATP8 exhibit the lowest and largest evolutionary rates, respectively [42,44].

### 4.2. Species Boundaries and Phylogenetics of the Three Dolycoris Species

*D. baccarum*, *D. indicus*, and *D. penicillatus* are distributed in the Chinese mainland with similar morphology. Previous studies on the three species have been limited to the descriptions of individual species, and the species boundaries and phylogenetic relationships among them are still unclear [17,18,19,20]. To clarify the species boundaries among the three *Dolycoris* species, we performed the analyses of species delimitation using four methods (ABGD, ASAP, bPTP, and GMYC) based on the animal DNA barcoding gene COI and the concatenated 13 PCGs. Most species delimitation analyses retrieved three OTUs, which corresponded well to the three *Dolycoris* species identified based on morphological characters. The bPTP analysis based on the concatenated 13 PCGs divided *D. penicillatus* into two OTUs, and this discordance between the bPTP and the morphological identification results may be due to the fact that the bPTP method tends to overestimate the species diversity when each population contains multiple sequences [28,48]. The distance-based method ABGD, based on COI and the concatenated 13 PCGs, assigned the individuals of *D. baccarum* and *D. penicillatus* to a single OTU, possibly due to the low genetic distance between *D. baccarum* and *D. penicillatus*. Compared to the interspecific genetic distance between *D. baccarum* and *D. indicus* and between *D. penicillatus* and *D. indicus*, the interspecific genetic distance between *D. baccarum* and *D. penicillatus* is lower, but significantly higher than the intraspecific genetic distances of the three *Dolycoris* species. A clear barcode gap has been found between the interspecific and intraspecific genetic distances in the three species of *Dolycoris*. We then reconstructed phylogenetic trees based on mitochondrial sequences to explore the phylogenetic relationships of these three *Dolycoris* species and their status within Pentatomidae. Our results indicate that the species of *Dolycoris* are nested within a clade consisting of Pentatominae species, with *Rubiconia intermedia* as their sister taxon, in agreement with the findings of a previous study [35]. Within *Dolycoris*, the phylogenetic relationship of *D. indicus* as a sister of *D. baccarum* + *D. penicillatus* was supported by most of the results except for the results of the BI tree based on the PCG123R dataset.

## 5. Conclusions

In this study, we performed mitogenome comparison, species delimitation, and phylogenetic analyses for three *Dolycoris* species. Our results indicate that the mitogenomes of the *Dolycoris* species are conserved in gene arrangement, nucleotide composition, and codon usage. The species boundaries of the three species were clarified, and a clear barcode gap was discovered between the interspecific and intraspecific genetic distances. Most of our phylogenetic results support the tree topology: (*D. indicus* + (*D. baccarum* + *D. penicillatus*)).

## Figures and Tables

**Table 1:** Length and nucleotide composition of the whole mitogenomes of the three *Dolycoris* species.

Species	Individuals	Length (bp)	T%	C%	A%	G%	A + T%	AT-Skew	GC-Skew
*D. baccarum*	HEBCZ	15,662	31.3	15.5	41.9	11.3	73.2	0.14	−0.16
	JSPD	15,474	31.3	15.6	41.9	11.3	73.2	0.14	−0.16
	JXLS	15,785	31.3	15.5	41.9	11.2	73.2	0.14	−0.16
	NMJS	15,554	31.3	15.5	41.9	11.3	73.2	0.14	−0.16
	QHQQ	15,484	31.3	15.5	41.9	11.3	73.2	0.14	−0.16
	SNZC	15,712	31.3	15.5	41.9	11.3	73.2	0.14	−0.16
*D. indicus*	YNLJ	15,686	31.3	15.5	41.7	11.5	73.0	0.14	−0.15
	YNNN	15,770	31.4	15.5	41.6	11.5	73.0	0.14	−0.15
	YNYY	15,255	31.2	15.6	41.8	11.4	73.0	0.15	−0.16
*D. penicillatus*	XJBS	15,630	31.2	15.6	41.9	11.3	73.1	0.15	−0.16
	XJHQ	15,526	31.2	15.6	41.9	11.3	73.1	0.15	−0.16
	XJTP	15,460	31.2	15.6	41.9	11.3	73.1	0.15	−0.16
	XJTS	15,508	31.2	15.6	41.9	11.3	73.1	0.15	−0.16
	XJTY	15,558	31.2	15.6	41.9	11.4	73.1	0.15	−0.16
	XJYB	15,558	31.2	15.6	41.9	11.3	73.1	0.15	−0.16

**Table 2:** Intraspecific and interspecific genetic distances of *Dolycoris* species inferred from single PCG and concatenated 13 PCGs. Abbreviations: DB, *Dolycoris baccarum*; DI, *Dolycoris indicus*; and DP, *Dolycoris penicillatus*.

Gene	Intraspecific Distance (%)			Interspecific Distance (%)		
	DB	DI	DP	DB-DI	DB-DP	DI-DP
ATP6	0.19	0.29	0.18	2.88	0.93	3.11
ATP8	0.43	0.87	0.60	3.78	3.32	6.13
COI	0.18	0.13	0.28	2.91	1.55	3.12
COII	0.27	0.10	0.29	3.12	0.79	2.70
COIII	0.29	0.00	0.25	2.20	0.96	2.34
CytB	0.38	0.06	0.11	3.71	1.23	3.16
ND1	0.34	0.07	0.28	1.92	1.34	1.77
ND2	0.20	0.07	0.14	2.44	1.32	2.50
ND3	0.36	0.00	0.29	3.16	2.32	2.67
ND4	0.22	0.10	0.22	2.95	0.97	3.09
ND4L	0.12	0.23	0.23	3.20	0.18	3.14
ND5	0.31	0.08	0.17	2.47	1.05	2.20
ND6	0.47	0.00	0.35	4.06	1.07	3.68
13 PCGs	0.27	0.10	0.22	2.84	1.20	2.78

## Data Availability

The data that support the findings of this study are openly available in GenBank (accession numbers: OQ909509–OQ909523) at https://www.ncbi.nlm.nih.gov/, accessed on 2 May 2023.

## Supplementary Materials

The following supporting information can be downloaded at: https://www.mdpi.com/article/10.3390/insects15020134/s1, Table S1. Sample information of *Dolycoris* species in the present study. Table S2. Taxonomic information and GenBank accession numbers of mitochondrial genomes downloaded from GenBank in this study. Table S3. The best model for each partition of PCG123R and PCG12R datasets. Table S4. Start and termination codons of PCGs in the mitogenomes of *Dolycoris*. Figure S1. Patterns of codon usage in the mitogenomes of the *Dolycoris*. The X-axis shows the codon families, and the Y-axis shows the total codons. Figure S2. Heterogeneity analysis of dataset PCG123R (A) and PCG12R (B). The mean similarity score between sequences is represented by a colored square, based on the AliGROOVE scores ranging from –1, indicating a large difference in rates from the remainder of the dataset (red coloration), to +1, indicating rates match all other comparisons (blue coloration). Figure S3. Phylogenetic trees of Pentatomidae inferred by BI analysis based on dataset PCG123R (A) and PCG12R (B). Numbers at the nodes are posterior probabilities.
