# Supplementary material for: Mitogenomes Provide Insights into the Species Boundaries and Phylogenetic Relationships among Three Dolycoris Sloe Bugs (Hemiptera: Pentatomidae) from China
Source: Insects. 2024 Feb 17;15(2):134. doi: 10.3390/insects15020134 (PMC10889809; doi:10.3390/insects15020134)
Supplement: Supplementary file 1 [file insects-15-00134-s001.zip › insects-2848885-Supporting information.pdf]

## Supporting Information

**Table S1.** Sample information of *Dolycoris* species in the present study.

| Species | Code | Locality | Longitude | Latitude | Collection date | Accession number |
|---|---|---|---|---|---|---|
| *D. baccarum* | HEBCZ | Zhengzhuangzi village, Caofeidian district, Hebei, China | 118.343 | 39.329 | 2020/6/6 | OQ909509 |
| *D. baccarum* | JSPD | Dishuizhu village, Pukou district, Jiangsu, China | 118.570 | 32.110 | 2019/7/17 | OQ909510 |
| *D. baccarum* | JXLS | Lu Mountain, Jiujiang city, Jiangxi, China | 116.009 | 29.557 | 2020/8/1 | OQ909511 |
| *D. baccarum* | NMJS | Shamujia town, Jiuyuan district, Neimenggu, China | 110.340 | 40.592 | 2017/8/18 | OQ909512 |
| *D. baccarum* | QHQQ | Qingyang Ditch, Qilian county, Qinghai, China | 100.402 | 38.149 | 2020/8/1 | OQ909513 |
| *D. baccarum* | SNZC | Chenjia Ditch, Zichang county, Shanxi, China | 109.618 | 36.459 | 2019/8/4 | OQ909514 |
| *D. indicus* | YNLJ | Jiuxi Mountain, Luxi county, Yunnan, China | 103.685 | 24.414 | 2020/7/29 | OQ909515 |
| *D. indicus* | YNNN | Ninger town, Ninger county, Yunnan, China | 101.054 | 23.034 | 2021/7/12 | OQ909516 |
| *D. indicus* | YNYY | Yulong village, Yulong county, Yunnan, China | 100.209 | 26.986 | 2022/7/25 | OQ909517 |
| *D. penicillatus* | XJBS | Sailimu Lake, Bole city, Xinjiang, China | 81.169 | 44.494 | 2016/8/6 | OQ909518 |
| *D. penicillatus* | XJHQ | Qingshuihe town, Huocheng county, Xinjiang, China | 80.731 | 44.203 | 2016/8/5 | OQ909519 |
| *D. penicillatus* | XJTP | Putao Ditch, Tulufan city, Xinjiang, China | 89.251 | 43.018 | 2016/7/28 | OQ909520 |
| *D. penicillatus* | XJTS | Tacheng Forest Park, Tacheng city, Xinjiang, China | 82.953 | 46.760 | 2016/8/10 | OQ909521 |
| *D. penicillatus* | XJTY | Yemenle town, Tacheng city, Xinjiang, China | 83.076 | 46.657 | 2016/8/9 | OQ909522 |
| *D. penicillatus* | XJYB | Balake village, Yining city, Xinjiang, China | 81.313 | 43.951 | 2016/8/4 | OQ909523 |

**Table S2.** Taxonomic information and GenBank accession numbers of mitochondrial genomes downloaded from GenBank in this study.

| Family | Subfamily | Species | Accession number |
|---|---|---|---|
| Pentatomidae | Asopinae | *Arma custos* | MT535604 |
| Pentatomidae | Asopinae | *Cazira horvathi* | NC_042817 |
| Pentatomidae | Asopinae | *Dinorhynchus dybowskyi* | NC_037724 |
| Pentatomidae | Asopinae | *Eocanthecona thomsoni* | NC_042816 |
| Pentatomidae | Asopinae | *Picromerus griseus* | NC_036418 |
| Pentatomidae | Asopinae | *Zicrona caerulea* | MW847250 |
| Pentatomidae | Pentatominae | *Carbula sinica* | KY069964 |
| Pentatomidae | Pentatominae | *Catacanthus incarnatus* | MF497716 |
| Pentatomidae | Pentatominae | *Caystrus obscurus* | MF497717 |
| Pentatomidae | Pentatominae | *Dalpada cinctipes* | MW847236 |
| Pentatomidae | Pentatominae | *Erthesina fullo* | JQ743673 |
| Pentatomidae | Pentatominae | *Eurydema dominulus* | MW847238 |
| Pentatomidae | Pentatominae | *Eurydema gebleri* | NC_027489 |
| Pentatomidae | Pentatominae | *Eurydema maracandica* | NC_037042 |
| Pentatomidae | Pentatominae | *Glaucias dorsalis* | MW847239 |
| Pentatomidae | Pentatominae | *Halyomorpha halys* | NC_013272 |
| Pentatomidae | Pentatominae | *Hippotiscus dorsalis* | MW847240 |
| Pentatomidae | Pentatominae | *Hoplistodera incisa* | MF620037 |
| Pentatomidae | Pentatominae | *Menida violacea* | MK617948 |
| Pentatomidae | Pentatominae | *Neojurtina typica* | MW847243 |
| Pentatomidae | Pentatominae | *Nezara viridula* | NC_011755 |
| Pentatomidae | Pentatominae | *Pentatoma metallifera* | MW847244 |
| Pentatomidae | Pentatominae | *Pentatoma semiannulata* | MT985377 |
| Pentatomidae | Pentatominae | *Placosternum urus* | MF497730 |
| Pentatomidae | Pentatominae | *Plautia fimbriata* | MF497731 |
| Pentatomidae | Pentatominae | *Plautia lushanica* | MW847245 |
| Pentatomidae | Pentatominae | *Rubiconia intermedia* | KP207596 |
| Pentatomidae | Phyllocephalinae | *Dalsira scabrata* | NC_037374 |
| Pentatomidae | Phyllocephalinae | *Gonopsis affinis* | NC_036745 |
| Pentatomidae | Podopinae | *Graphosoma rubrolineatum* | NC_033875 |
| Pentatomidae | Podopinae | *Scotinophara lurida* | NC_042815 |
| Plataspidae | | *Megacopta cribraria* | OP123001 |
| Plataspidae | | *Calacta lugubris* | NC_058965 |

**Table S3.** The best model for each partition of PCG123R and PCG12R datasets.

| Datasets | Partition names | Best Model |
|---|---|---|
| PCG123R | ATP6, CO1, CO2, CO3, CytB, ND3; | GTR+I+G |
| | ATP8, ND2, ND6; | GTR+I+G |
| | ND1, ND4, ND4L, ND5, 12S, 16S. | TIM+I+G |
| PCG12R | ATP6, ATP8, ND1, ND2, ND3, ND4L, ND6; | GTR+I+G |
| | CO1, CO2, CO3, CytB; | GTR+I+G |
| | ND4, ND5, 12S, 16S. | TIM+I+G |

**Table S4.** Start and termination codons of PCGs in the mitogenomes of *Dolycoris*.

| Species | Individuals | ATP6 | ATP8 | COI | COII | COIII | CytB | ND1 | ND2 | ND3 | ND4 | ND4L | ND5 | ND6 |
|---|---|---|---|---|---|---|---|---|---|---|---|---|---|---|
| | | **Start codon** | | | | | | | | | | | | |
| *D. baccarum* | HEBCZ | ATG | ATA | TTG | ATA | ATG | ATG | ATA | ATT | ATA | ATG | ATT | ATA | ATA |
| *D. baccarum* | JSPD | ATG | ATA | TTG | ATA | ATG | ATG | ATA | ATT | ATA | ATG | ATT | ATA | ATA |
| *D. baccarum* | JXLS | ATG | ATA | TTG | ATA | ATG | ATG | ATA | ATT | ATA | ATG | ATT | ATA | ATA |
| *D. baccarum* | NMJS | ATG | ATA | TTG | ATA | ATG | ATG | ATA | ATT | ATA | ATG | ATT | ATA | ATA |
| *D. baccarum* | QHQQ | ATG | ATA | TTG | ATA | ATG | ATG | ATA | ATT | ATA | ATG | ATT | ATA | ATA |
| *D. baccarum* | SNZC | ATG | ATA | TTG | ATA | ATG | ATG | ATA | ATT | ATA | ATG | ATT | ATA | ATA |
| *D. indicus* | YNLJ | ATG | ATA | TTG | ATA | ATG | ATG | ATA | ATT | ATA | ATG | ATT | ATA | ATA |
| *D. indicus* | YNNN | ATG | ATA | TTG | ATA | ATG | ATG | ATA | ATT | ATA | ATG | ATT | ATA | ATA |
| *D. indicus* | YNYY | ATG | ATA | TTG | ATA | ATG | ATG | ATA | ATT | ATA | ATG | ATT | ATA | ATA |
| *D. penicillatus* | XJBS | ATG | ATA | TTG | ATA | ATG | ATG | ATA | ATT | ATA | ATG | ATT | ATA | ATA |
| *D. penicillatus* | XJHQ | ATG | ATA | TTG | ATA | ATG | ATG | ATA | ATT | ATA | ATG | ATT | ATA | ATA |
| *D. penicillatus* | XJTP | ATG | ATA | TTG | ATA | ATG | ATG | ATA | ATT | ATA | ATG | ATT | ATA | ATA |
| *D. penicillatus* | XJTS | ATG | ATA | TTG | ATA | ATG | ATG | ATA | ATT | ATA | ATG | ATT | ATA | ATA |
| *D. penicillatus* | XJTY | ATG | ATA | TTG | ATA | ATG | ATG | ATA | ATT | ATA | ATG | ATT | ATA | ATA |
| *D. penicillatus* | XJYB | ATG | ATA | TTG | ATA | ATG | ATG | ATA | ATT | ATA | ATG | ATT | ATA | ATA |
| | | **Termination codon** | | | | | | | | | | | | |
| *D. baccarum* | HEBCZ | TAA | TAA | T | T | TAA | TAA | TAA | TAA | TAA | TAA | TAA | TAA | TAA |
| *D. baccarum* | JSPD | TAA | TAA | T | T | TAA | TAA | TAA | TAA | TAA | TAA | TAA | TAA | TAA |
| *D. baccarum* | JXLS | TAA | TAA | T | T | TAA | TAA | TAA | TAA | TAA | TAA | TAA | TAA | TAA |
| *D. baccarum* | NMJS | TAA | TAA | T | T | TAA | TAA | TAA | TAA | TAA | TAA | TAA | TAA | TAA |
| *D. baccarum* | QHQQ | TAA | TAA | T | T | TAA | TAA | TAA | TAA | TAA | TAA | TAA | TAA | TAA |
| *D. baccarum* | SNZC | TAA | TAA | T | T | TAA | TAA | TAA | TAA | TAA | TAA | TAA | TAA | TAA |
| *D. indicus* | YNLJ | TAA | TAA | T | T | TAA | TAA | TAA | TAA | TAA | TAA | TAA | TAA | TAA |
| *D. indicus* | YNNN | TAA | TAA | T | T | TAA | TAA | TAA | TAA | TAA | TAA | TAA | TAA | TAA |
| *D. indicus* | YNYY | TAA | TAA | T | T | TAA | TAA | TAA | TAA | TAA | TAA | TAA | TAA | TAA |
| *D. penicillatus* | XJBS | TAA | TAA | T | T | TAA | TAA | TAA | TAA | TAA | TAA | TAA | TAA | TAA |
| *D. penicillatus* | XJHQ | TAA | TAA | T | T | TAA | TAA | TAA | TAA | TAA | TAA | TAA | TAA | TAA |
| *D. penicillatus* | XJTP | TAA | TAA | T | T | TAA | TAA | TAA | TAA | TAA | TAA | TAA | TAA | TAA |
| *D. penicillatus* | XJTS | TAA | TAA | T | T | TAA | TAA | TAA | TAA | TAA | TAA | TAA | TAA | TAA |
| *D. penicillatus* | XJTY | TAA | TAA | T | T | TAA | TAA | TAA | TAA | TAA | TAA | TAA | TAA | TAA |
| *D. penicillatus* | XJYB | TAA | TAA | T | T | TAA | TAA | TAA | TAA | TAA | TAA | TAA | TAA | TAA |

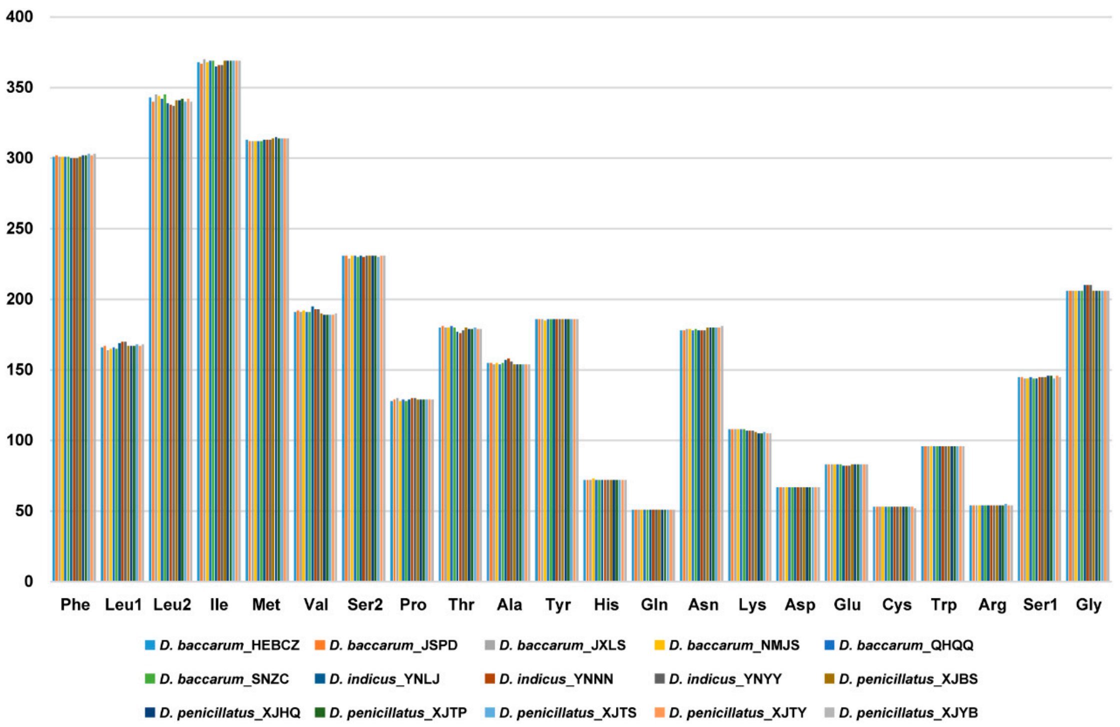


**Figure S1.** Patterns of codon usage in the mitogenomes of the *Dolycoris*. The X-axis shows the codon families and the Y-axis shows the total codons.

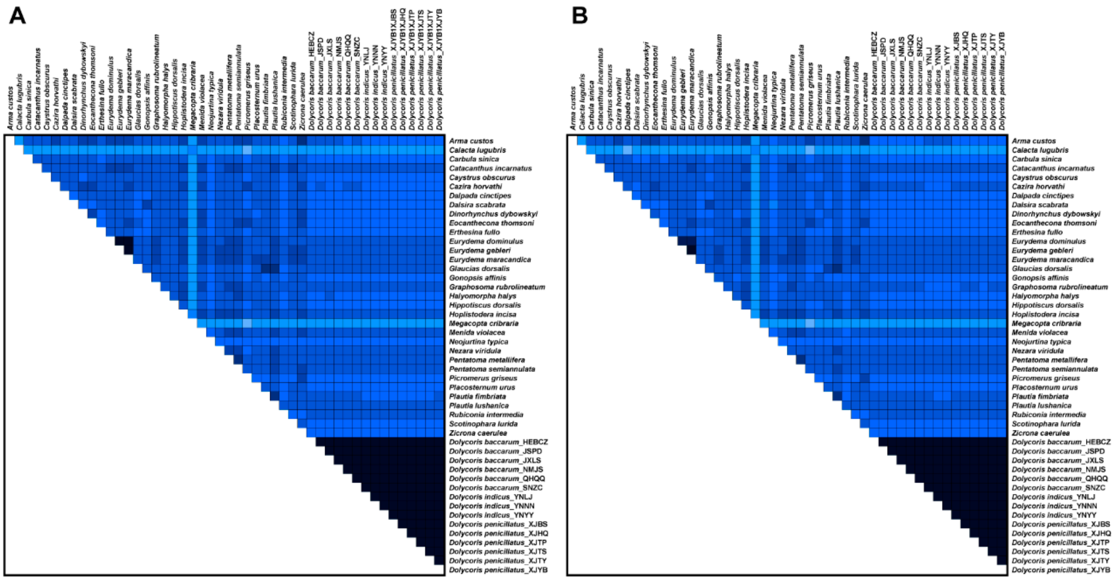


**Figure S2.** Heterogeneity analysis of dataset PCG123R (A) and PCG12R (B). The mean similarity score between sequences is represented by a colored square, based on the AliGROOVE scores ranging from –1, indicating a large difference in rates from the remainder of the dataset (red coloration), to +1, indicating rates match all other comparisons (blue coloration).

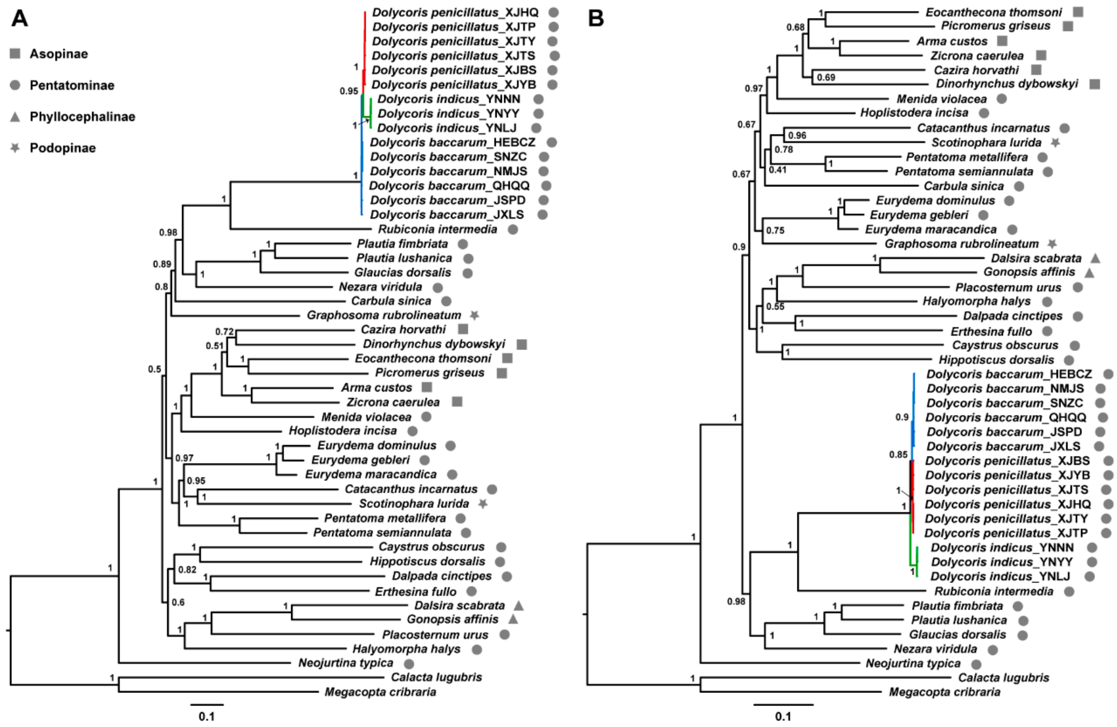


**Figure S3.** Phylogenetic trees of Pentatomidae inferred by BI analysis based on dataset PCG123R (A) and PCG12R (B). Numbers at the nodes are posterior probabilities.
